# The Importance of Mental Well-Being for Health Professionals During Complex Emergencies: It Is Time We Take It Seriously

**DOI:** 10.9745/GHSP-D-17-00017

**Published:** 2017-06-27

**Authors:** Mary Surya, Dilshad Jaff, Barbara Stilwell, Johanna Schubert

**Affiliations:** a University of North Carolina at Chapel Hill, School of Nursing, Chapel Hill, NC, USA.; b University of North Carolina at Chapel Hill, Gillings School of Global Public Health, Chapel Hill, NC, USA.; c IntraHealth International, Chapel Hill, NC, USA.

## Abstract

We call on humanitarian aid organizations to integrate proven mental health strategies to protect the mental health of their workforce and improve staff capacity to provide care for vulnerable populations. Such strategies could include:

- Pre-deployment training
- Art therapy
- Team building
- Physical exercise
- Mindfulness or contemplative techniques
- Mind-body exercises
- Narrative Exposure Therapy
- Eye movement desensitization and reprocessing

## BACKGROUND

The number of people displaced from their homes or villages as a result of conflict has increased in the last 20 years.^1^ As a result, millions have been forced to live far from their homes and communities in refugee camps sometimes for months or years. In 2014, an estimated 60 million refugees worldwide fled war-torn and conflict areas and, of these, more than 60% were forcibly uprooted and displaced within their own countries, according to the United Nations High Commissioner for Refugees (UNHCR).^1^ As a result, the demand for an emergency health care response has increased, as has the need for a qualified health care workforce, particularly nurses, physicians, and similarly trained or licensed professionals.^2^ Additionally, as the number of manmade and natural disasters have increased, the demand for aid has also risen, especially in settings where complex emergencies have occurred. The World Health Organization (WHO) defines complex emergencies as “combin[ing] internal conflict with large-scale displacements of people, mass famine or food shortage, and fragile or failing economic, political, and social institutions. Often, complex emergencies can be created by natural disasters.”^3^ With the incidence of global complex emergencies and humanitarian crises rising, local and expatriate health professionals have become increasingly exposed to stress and trauma for protracted periods. This type of stress and psychological trauma can be further defined as primary or secondary: primary stress and psychological trauma involves direct dangers or events that happen to one's self while secondary stress and trauma results from exposure to the experiences of others.^4^

With the rising incidence of global complex emergencies and humanitarian crises, health professionals have become increasingly exposed to stress and trauma for protracted periods.

While small steps have been taken to mitigate mental health consequences, more can be done to support the psychosocial well-being of health professionals in crisis situations. This article calls on humanitarian aid organizations to address the mental health of their local and expatriate workforce by integrating proven mental health strategies, including psychosocial support, into health delivery models, thereby protecting, enhancing, and improving staff capacity to provide care for vulnerable populations. In this article, the authors review the possible effects of acute and prolonged stress on the mental health of health professionals, and propose new ways for organizations to ensure that health professionals have the support they need in order to remain effective in the field.

## COMPLEX EMERGENCIES AND THEIR CONSEQUENCES ON HEALTH PROFESSIONALS

WHO defines mental health as “a state of well-being in which the individual realizes his or her own abilities, can cope with the normal stresses of life, can work productively and is able to make a contribution to his or her community.”^5^ The mental and psychological well-being of health professionals is imperative to their ability to function effectively, particularly when exposed to extreme environments. Such exposure could result in negative mental health consequences, which may in turn affect the functioning and productivity of humanitarian organizations.^6^ Health professionals, particularly those working in complex and rapidly changing environments, experience high levels of stress due to overwhelming work demands such as long hours, extreme temperatures, unsafe and unfamiliar environments, and exposure to violence and human suffering.^7^^–^^10^

Acute episodes of stress can have the beneficial effect of enhancing our reactions and abilities; however, it can also lead to further mental and physical distress. “Stress is positive when it forces us to adapt and thus to increase the strength of our adaptation mechanisms, warns us that we are not coping well and that a lifestyle change is warranted if we are to maintain optimal health.”^11^ Distress occurs when a stressful or disturbing situation exceeds a person's ability to process it in a healthy way. It can be debilitating^12^ and can lead to depression, fatigue, depersonalization, and burnout.^8^ Posttraumatic stress disorder (PTSD) occurs when a person who has experienced a shocking or dangerous event continues to suffer clinical symptoms—such as intrusive memories and flashbacks, avoidant behavior, and hyperarousal—beyond the first 4 weeks after the traumatic event has passed.^13^ Studies have also shown that health professionals working in extreme settings are vulnerable to symptoms of PTSD as a reaction to assisting traumatized and distressed individuals.^14^ This form of secondary traumatic stress—also known as “compassion fatigue” or “vicarious trauma”—usually develops over time through prolonged indirect exposure to traumatic events, such as narratives of firsthand experiences.^15^^,^^16^ Organizationally, both types of stress can contribute to decreased efficiency and effectiveness.^17^

Acute episodes of stress can have the beneficial effect of enhancing our reactions and abilities, but it can also lead to further mental and physical distress.

Evidence shows that chronic stress and posttraumatic stress have serious health consequences.^18^^–^^20^ Chronic stress can alter the immune system, resulting in immune suppression or excessive inflammatory reactions.^11^^,^^21^ Stress impacts the immune system through multiple physiological pathways and contributes to chronic illness. Stress can alter sleep patterns and lead to behavioral changes that further influence health, such as using drugs or tobacco or using excessive amounts of alcohol or caffeine.^22^ Physical illnesses associated with or exacerbated by stress, include asthma,^23^ gastroesophageal reflux disease,^24^ irritable bowel syndrome,^25^ peptic ulcer disease,^26^ coronary artery disease,^27^ myocardial infarction,^28^ and cancer.^29^ Chronic stress and psychological distress may also result in physical pain or other illness such as depression and anxiety.^30^^,^^31^ Health professionals continue to be at increased risk for depression and burnout after they return from deployments, and studies have found that this risk continues even up to 3 to 6 months after assignment completion.^6^ On the basis of the data presented, it is plausible that such factors may affect all those impacted by complex emergencies. Therefore, in order to provide effective interventions in these settings, it is crucial for humanitarian aid organizations to prioritize mental well-being for their staff at both organizational and operational levels.

## EPIDEMIOLOGY

Some studies suggest that 70% to 89% of humanitarian aid workers have experienced mental health issues related to their job.^6^^,^^17^^,^^32^ However, researchers noted that “the majority of agencies contacted from the initial list of possible organizations declined participation or did not respond to the inquiry,”^6^ suggesting that these figures may not adequately measure the scope of the problem. Research focusing on national health staff engaged in aid work in China found that 30% of national Red Cross nurses experienced PTSD symptoms compared with 10.2% of their nursing peers not involved in disaster relief.^33^ Many tools have been developed to measure symptoms of psychological distress, such as anxiety, depression, burnout, emotional exhaustion, depersonalization, substance use disorder, and life or job dissatisfaction. However, just as there is no universal expression of distress, even validated tools for depression and anxiety cannot assess fully and accurately its prevalence. Furthermore, simply identifying and assessing current needs poses a challenge because most stress and distress levels among the workforce are self-reported, but not systematically so. Literature indicates that health professionals who have worked in high-risk or high-stress situations are not tracked after service, which means that self-reporting is most likely the way that ongoing mental health needs would be known or met.^10^ According to a survey carried out by UNHCR among its own staff, only one-quarter of the staff who had suffered a life-threatening critical incident had any form of longer-term follow up. UNHCR recommended investment in an interactive confidential website, which would provide access to a self-assessment tool and Skype consultation with an external therapist.^17^

70% to 89% of humanitarian aid workers have experienced mental health issues related to their job, according to some studies.

## FAILURE TO IMPLEMENT CURRENT GUIDELINES

Introducing systems and processes to support evidence-based practices on an organizational level is a priority for recognizing and addressing the psychosocial needs of health professionals and members of the populations affected by crisis. The Inter-Agency Standing Committee (IASC) has developed guidelines for organizations to address the mental health and psychosocial support needs of their workforce. The guidelines outline many key actions, including^34^:

- Ensuring availability of a concrete plan to protect and promote staff well-being, specific to the emergency
- Ensuring national and international staff receive adequate information and training in the emergency context to prepare them for their role
- Addressing work-related stressors
- Ensuring access to health care and psychosocial support
- Providing support, such as psychological first aid, to staff for all critical incidents
- Ensuring availability of post-deployment support

Some organizations have policies in place similar to *UNHCR's Mental Health and Psychosocial Support for Staff*, which outlines the need for psychosocial support and highlights 8 principles of good practice.^17^ In addition, the Anteres Foundation's *Managing Stress in Humanitarian Aid Workers, Guidelines for Good Practice* identified key principles for organizations, including policy, screening and assessment, preparation and training, monitoring, ongoing support, crisis support, end-of-assignment support, and post-assignment support.^35^ However, according to surveys conducted among several humanitarian aid agencies, there was only 35% to 68% compliance with these principles organizationally.^17^ While international guidelines on addressing the mental health and psychosocial support needs of humanitarian aid workers are in place, they are not being successfully implemented.

While international guidelines on addressing the mental health and psychosocial support need of humanitarian aid workers are in place, they are not being successfully implemented.

## ADDRESSING THE MOST PRESSING CHALLENGES

Four of the most pressing challenges for implementation of mental health interventions for health professionals in complex settings include (1) mental health stigma around relief work, (2) limited organizational capacity to provide the necessary mental health support for their staff, (3) structural inequalities, and (4) lack of mental health services in mission countries and globally.

### Mental Health Stigma and Attitudes Around Relief Work

In some cases, a culture of “heroism” surrounds relief and rescue work. Because the construct is based on a mission of helping others, it perpetuates the myth that the “heroes” offer help but do not require any support for themselves.^36^ Institutionally, this stance may be reinforced as the overwhelming volume of work limits the time and resources available for self-reflection and processing of difficult situations. An attitude of self-sacrifice may also creep into the ego of many relief workers leading to a discomfort with being physically and emotionally comfortable while others are suffering. As one field-based health professional notes in her blog, “I have come to see that when I am faced with a waiting room full of patients who themselves are likely hungry, it is really OK to break for a cup of tea and an oatmeal nut bar, if that is what it takes to keep me ‘buoyant’.”^37^ Through this experience, the writer realized that in taking time to address her own physical health she would be better able to help others. In order to develop effective mental health interventions for health professionals, the range of stigma and attitudes around relief work should be acknowledged and futher examined.

### Organizational Capacity

Many NGOs lack appropriate support measures for their staff.^38^ For some NGOs, support and access may be limited by financial constraints, resulting in a near absence of effective formal mental health services, especially in countries where complex emergencies occur. Access is also limited by the poor integration of psychosocial support into the structure of many humanitarian organizations. Even where staff welfare services exist, utilization is not always guaranteed due to employees' lack of time, skepticism about trustworthiness and confidentiality, or resistance to admit the need for help.^39^^,^^40^ Understanding the protective factors and coping mechanisms available to health professionals is important. Many resources have been reported as possible moderators of or risk factors for the effects of chronic and traumatic stress during deployment. Social support has been identified as a moderator of the effects of trauma exposure leading to PTSD for humanitarian workers^41^ and inadequate communication with family and friends has been identified as a risk factor for depression.^42^^,^^43^ Another factor related to the development of depression among health workers is lack of support from within the aid organization.^42^ This lack of support is compounded by the fact that communication in complex emergencies is unreliable at best and reaching outside the country and organization may not be an option for individuals seeking support.

### Structural Inequalities

**Inequality between staff members.** While the entire health workforce can experience stress and distress, those who have known personal injustice may be at the highest risk. Health workers who have experienced paternalism, harassment, racism, homophobia, religious persecution, or any other perceived injustice may be predisposed to feeling overwhelmed or traumatized when they witness abuse during a field assignment. As was pointed out in the IASC guidelines, “emergencies erode normally protective supports, increase the risk of diverse problems and tend to amplify pre-existing problems of social injustice and inequality.”^34^ This is as much true for the population affected by the emergency as it is for the health care responders.

Taking gender as an example, research on demographic risk factors for PTSD in the general population suggests that women are twice as likely as men to develop this condition in their lifetime.^44^ Being a woman also increases the risk of developing depression and anxiety.^45^ These risks can be exacerbated when working in complex and stressful settings with no support. Contributing to these statistics is likely the experience women have in their workplaces. For example, responsibility without power is likely to be highly stressful, and this situation can happen when organizations fail to offer women equity within positions of power or adequate levels of decision making. Moreover, organizations may fail to protect the interest or safety of women who are at risk of experiencing paternalism, harassment, exploitation, violence, or abuse resulting from the cultural subordination of women. Without the opportunity for self-reflection, a systemized means to report abuse or discrimination, or the time for perceived injustices to be respectfully acknowledged, women may experience higher levels of distress due to a lack of adequate means of support within their organization. Research in this area is scarce.

**Opportunities for staff feedback.** Without feedback, organizations are left without the information or pressure needed to examine and adjust their policies and structure. Due to the increasing trend of short-term contracts in international organizations, physically and emotionally exhausted staff might drop out at the end of their term for reasons unnoticed by their organizations, as few organizations assess the reasons why short-term staff do not not reapply for positions. Ideally, aid workers' social networks and health systems will help with psychosocial aftercare once they leave the organization or sector. Currently, no data are available to show what percentage of international humanitarian workers leave the sector for psychosocial reasons, or how long individuals need, on average, to recover before taking on a new position or occupation. Even if organizations receive feedback, those without a formal process to respond and implement change may be hesitant to acknowledge grievances for fear of being discredited and losing contracts. For example, if concerns by any one group are not being addressed it is likely to decrease motivation and productivity, thus limiting the ability of the organization to effectively accomplish their objectives and mission. This lack of feedback may also perpetuate structural inequalities within the organization by leaving the concerns unrecognized or unaddressed. Ultimately, this may affect the organization's credibility and decrease its chances for future funding or qualified applicants. When an organization is one of the few available to offer support during a crisis, then it may seem especially challenging to dedicate time or resources to address structural inequalities.

### Lack of Mental Health Services in Mission Countries and Globally

Formal mental health services in low- and middle-income countries are almost nonexistent. There are only 9 mental health providers per 100,000 people globally; an extra 1.7 million mental health workers are needed in low- and middle-income countries alone.^46^ Even in wealthy countries, 40% to 60% of people with severe mental disorders do not receive adequate care.^47^ Despite the lack of resources, lessons may be learned from countries where official mental health services are not yet fully functioning but informal community-level psychological support has been implemented. Well-organized psychological support at the community level can be particularly effective because it is usually adapted to meet a community's needs and resources. However, for an international health worker with a unique spectrum of personal experience, this may make care especially difficult to access or even understand, depending on the specific culture of mental health care. Additionally, when social structures break down due to a local crisis, implementation or reconstruction of informal support systems may be more difficult, and perceptions may be that such services are for local beneficiaries, rather than international aid workers.

Formal mental health services in low- and middle-income countries are almost nonexistent, and even in wealthy countries, 40% to 60% of people with severe mental disorders do not receive adequate care.

## STRATEGIES, TOOLS, AND RESOURCES

Strengthening the capacity for resilience is key to mental health for global health professionals and local populations alike. Resilience, in this context, is the ability to adapt and respond to changing circumstances while maintaining physical and emotional wellness. Several specific evidence-based practices have been successful to reduce or mitigate distress; decrease specific trauma-related symptoms, such as anxiety, depression, PTSD, or burnout; and improve resilience among health workers or others involved in complex emergencies. Some practices can be used for self-support after training, while others should be used by therapists with specialist training:

- **Pre-deployment training** is a way of offering preparation for unexpected or unsettling situations that may arise during a particular health care response to disaster and providing opportunities for pre-deployment self-reflection and awareness of potential vulnerabilities.^48^ Psychological first aid manuals and a number of other well-organized guides are available, including *Psychological First Aid: Facilitator's Manual for Orienting Field Worker*s.^49^
- **Team building** involves strengthening communication and relationships and enhancing a sense of belonging between coworkers, which may result in improved delivery of care and overall outcomes.^50^ Building a sense of belonging in the workplace, specifically, has been shown to be protective against organizational stressors and is an important strategy in the cultivation of resilience.^50^
- **Mindfulness or contemplative techniques**, such as Mindfulness-Based Stress Reduction (MBSR) practices and Progressive Muscle Relaxation,^51^ use stress reduction techniques based on mindfulness and (self) compassion and include practices such as body scan and sitting meditation.^52^ Relaxation response training is another technique that has been proven to help reduce stress and improve health.^53^
- **Narrative Exposure Therapy** (NET) is based on principles of behavioral and cognitive therapy and includes a wide range of activities, such as motivational interviewing, storytelling for resilience, and reflecting on and reconceptualizing traumatic experiences.^54^ NET has been used in the context of natural disasters to successfully treat symptoms including PTSD, anxiety, depression, general mental stress, and increased posttraumatic growth.^55^
- **Art therapy** involves a range of techniques that have been proven to enhance creativity, flexibility, problem solving, and coping skills, making this style of therapy a good fit for relief workers facing uncertain circumstances.^56^ One study showed similar outcomes for art therapy as a cognitive behavioral intervention for stress and trauma.^57^ Art therapy has also been used successfully among social workers in the context of war, suggesting that it would be feasible in complex or dangerous situations.^58^
- **Physical exercise** can be particularly problematic for people working very long exhausting hours, especially in settings with temperature extremes and limited access to safe space. Activities that involve minimal equipment such as jump roping, hula hooping, and dance, have been shown to reduce stress, burnout, and compassion fatigue.^59^^,^^60^
- **Mind-body exercises** are deliberate muscle movements combined with breathing and mental focus. Specific techniques, such as yoga^61^ and Tai chi,^62^ can both be effective against the ill effects of stress and build resilience.
- **Event-triggered counseling sessions** using techniques such as cognitive behavioral therapy, either in-person or using a telemedicine approach, attempt to challenge negative thoughts and promote resilience through cognitive flexibility and restructuring.
- **Eye movement densensitization and reprocessing** is a highly effective therapeutic short-term intervention to process both fresh and old traumatic experiences on the level of memory networks and the limbic system.^63^^,^^64^

## IDENTIFYING INNOVATIVE SOLUTIONS

Design thinking is “a systematic innovation process that prioritizes deep empathy for end-user desires, needs and challenges to fully understand a problem in hopes of developing more comprehensive and effective solutions.”^65^ Any psychosocial intervention developed to address distress among health professionals could benefit from integrating design-thinking strategies by, for example, integrating open-ended questions into the structure of an intervention and ensuring the program has the flexibility to react to feedback. Feedback questions might include: What does it look like when you feel stressed? Which parts or components of your day are the most stressful? What coping or relaxation strategies are effective for you? How might this organization support your coping techniques? A design-thinking approach allows organizations the opportunity to shift from problem-based single-solution thinking to a more interactive process-based style—providing program designers with the tools to address diverse experiences, expressions, and manifestations of stress and aid with coping and healing strategies. Design-thinking techniques can also be used to facilitate individual feedback and organizational reactivity, allow for opportunities to customize psychosocial support practices, and “improve capacity for uncovering unforeseen changes and unintended consequences.”^66^ Innovative feedback mechanisms also have the potential to offer opportunities to explore and address structural inequalities within an organization in manageable ways. Finally, the quality of management on all organizational levels plays a crucial role in creating an enabling environment for stress prevention and recovery from high-intensity deployments, and creates a validating culture where staff feel empowered to talk about their personal challenges, including organizational shortcomings, without fear of consequences to future employment.

## DISCUSSION

The significant increase in complex global emergencies has resulted in prolonged stressful conditions for many health workers as (and after) they respond to humanitarian operations. While it is clear that chronic stress and distress are unhealthy and unsustainable, this review reveals that relief workers are not routinely provided with the adequate psychosocial support, tools, and strategies they need in order to be effective and remain healthy. While coping methods emerge naturally in response to stress and distress, not all responses are healthy. The literature demonstrates that chronic high levels of stress and distress contribute to *unhealthy* coping methods, resulting in poor health and decreased overall efficiency. Even though it is clearly in the interest of humanitarian organizations to provide support to health professionals who are stressed, we have found that such care is still scarce. The basic aims of supporting the health workforce under difficult circumstances includes cultivating more responsible engagement in fieldwork and encouraging cooperation, creativity, adaptability, and teamwork. Another aim of psychosocial support is to help health care providers develop their skills to become more focused practitioners and compassionate healers. Resilience and relaxation training, as well as psychosocial support, is also beneficial for organizations, particularly with regard to increased retention and enhanced productivity.

Chronic high levels of stress and distress contribute to unhealthy coping methods, resulting in poor health and decreased efficiency.

Organizations working in this context must prioritize psychosocial support and ensure that it is built into each component of their infrastructure:

- Screening and assessing (Many psychological screening tools are currently available but not widely or systematically used.)
- Preparation and training
- Monitoring
- Ongoing support
- Crisis support
- End-of-assignment support
- Post assignment support
- Policy framework

Strong organizational support has the potential to strengthen mental health and well-being during the process of rebuilding after a crisis. Using local mental health resources in field assignments is an admirable way to provide psychosocial support during and after a crisis or disaster. Unfortunately, use of local mental health resources may not always feasible due to poor or insufficient levels of services, disabled services as a result of a crisis, the types of services or infrastructure being targeted, safety and security concerns causing inaccessibility of services, or cultural, religious, and linguistic barriers.^67^ The need for more focused actions and joint opportunities tailored to psychosocial needs has never been greater. The Box summarizes strategies to address key challenges to improving the well-being of health professionals during complex emergencies.

BOX **Strategies to Improve Mental Well-Being of Health Professionals During Complex Emergencies for Organizations Working in These Settings**

- Train all expatriate and local staff on mental health first aid and selected peer supporters in counseling
- Standardize methods for prevention, reporting, and referral
- Recognize and address mental health issues
- Organize various activities to raise awareness about mental health issues, such as online courses, workshops, and trainings
- Explore opportunities within the existing local health or public service sector and strengthen (or build) those services
- Consider community-based training and intervention for local and international staff
- Conduct studies and systematic research to understand the size of the problem and to develop effective mechanisms
- Adapt and use available resources and techniques
- Improve and increase information sharing and communication related to mental health and well-being between organizations
- Set up comprehensive and supervised peer support systems to provide low-threshold contact points for affected staff members
- Consider the use of design-thinking techniques to integrate individual perspectives with organizational structure and to improve organizational response, flexibility, and adaptation.

## CONCLUSION

Globally, human safety and security will continue to be affected by ongoing complex emergencies. While the information and tools needed to improve the quality of psychosocial support for health professionals, and those with whom they interact, in these complex situations are available, substantial gaps related to mental health support for these health professionals still exist. Organizations and agencies must integrate these strategies into relief efforts, in order to improve the health, resilience, responsibility, creativity, and effectiveness of the workforce, while at the same time building organizational capacity to address organizations with whom they work. The time has come to address these issues by integrating psychosocial support into health delivery models for the health workforce. Doing so will help to protect the health of the workforce and improve their capacity to provide optimal care for the world's most vulnerable people.

## Notes

**Peer Reviewed**
